# Characterization of drug-induced liver injury associated with drug reaction with eosinophilia and systemic symptoms in two prospective DILI registries

**DOI:** 10.1007/s00204-023-03630-0

**Published:** 2023-12-05

**Authors:** Inmaculada Medina-Cáliz, Judith Sanabria-Cabrera, Marina Villanueva-Paz, Lauryna Aukštikalnė, Camilla Stephens, Mercedes Robles-Díaz, José M. Pinazo-Bandera, Miren García-Cortes, Isabel Conde, German Soriano, Fernando Bessone, Nelia Hernandez, Vinicius Nunes, Raymundo Paraná, M. Isabel Lucena, Raúl J. Andrade, Hao Niu, Ismael Alvarez-Alvarez

**Affiliations:** 1 Servicios de Aparato Digestivo y Farmacología Clínica, Hospital Universitario Virgen de La Victoria, Instituto de Investigación Biomédica de Málaga y Plataforma en Nanomedicina-IBIMA Plataforma BIONAND, Universidad de Málaga, Málaga, Spain; 2 Centro de Investigación Biomédica en Red Enfermedades Hepáticas y Digestivas (CIBERehd), Madrid, Spain; 3 Plataforma de Investigación Clínica y Ensayos Clínicos, Plataforma ISCIII de Investigación Clínica, UICEC-IBIMA, Madrid, Spain; 4 Institute of Physiology and Pharmacology, Lithuanian University of Health Sciences, Kaunas, Lithuania; 5 Unidad de Hepatología, Hospital Universitari I Politècnic La Fe, Servicio de Aparato Digestivo, Valencia, Spain; 6 Servicio de Gastroenterología, Hospital de La Santa Creu I Sant Pau, Universitat Autònoma de Barcelona, Barcelona, Spain; 7 Hospital Provincial del Centenario, Rosario, Argentina; 8 Hospital de Clínicas, Montevideo, Uruguay; 9 Hospital Universitário Prof. Edgard Santos-UFBA, Salvador, Brazil; 10 Departamento de Farmacología, Facultad de Medicina, Universidad de Málaga, Boulevard Louis, Pasteur 32, 29010 Málaga, Spain

**Keywords:** Drug-induced liver injury, Drug reaction with eosinophilia and systemic symptoms, Severe cutaneous adverse reaction, Hepatotoxicity, Hypersensitivity features

## Abstract

Idiosyncratic drug-induced liver injury (DILI) associated with drug reactions with eosinophilia and systemic symptoms (DRESS) is poorly characterized among patients of Western countries. We aimed to comprehensively assess the clinical characteristics, outcomes, and causative agents in a prospective, well-vetted cohort of DILI patients with DRESS (DILI-DRESS). We identified 53 DILI-DRESS cases from the Spanish DILI Registry and the Latin American DILI Network. For comparison purposes, we defined a group of DILI patients (n = 881). DILI-DRESS cases were younger (47 vs. 53 years, respectively; p = 0.042) and presented more frequently with cholestatic/mixed damage (p = 0.018). Most DILI-DRESS patients showed moderate liver injury, 13% developed severe damage, and only one patient (with hepatocellular injury due to anti-tuberculosis drugs) progressed to acute liver failure and died. DILI-DRESS cases showed a distinctive causative drug pattern compared to DILI cases. The most frequent drugs were carbamazepine (13%), anti-tuberculosis drugs (13%), amoxicillin-clavulanate (11%), and allopurinol and lamotrigine (7.6% each). Among all cases of DILI due to allopurinol and lamotrigine, 67% presented with a DILI-DRESS phenotype, respectively. Higher total bilirubin (TBL) levels at DILI recognition (odds ratio [OR] 1.23; 95% confidence interval [CI] 1.04–1.45) and absence of eosinophilia (OR 8.77; 95% CI 1.11–69.20) increased the risk for developing a severe-fatal injury in DILI-DRESS patients. DILI-DRESS patients have a more frequent cholestasis/mixed pattern of injury at presentation, with antiepileptics as distinctive causative drug class. Most of the lamotrigine and allopurinol cases present with this phenotype. Higher TBL levels and absence of eosinophilia at DILI recognition are markers of poor outcomes.

**Supplementary Information:**

The online version contains supplementary material available at 10.1007/s00204-023-03630-0.

## Introduction

Idiosyncratic drug-induced liver injury (DILI) is an unexpected reaction to conventional medications, herbal products, or dietary supplements (Andrade et al. 2019). From 14 to 25% of DILI cases courses with immunoalergic characteristics (Chalasani et al. 2015; Stephens et al. 2021) and, in this context, DILI can manifest with severe cutaneous adverse reactions (SCARs).

Drug Reaction with Eosinophilia and Systemic Symptoms (DRESS) is a challenging immune-mediated reaction caused by a variety of drugs that presents with dermatological manifestations, often as urticated maculopapular eruption (MPE), and systemic features, being the liver the most frequent internal organ involved. DRESS can progress from mild damage to fatal cases, with estimated mortality that ranges from 1.7 to 8% (Kardaun et al. 2013; Lee et al. 2022). Previous studies have identified limited drugs and drug classes as responsible agents for DRESS, such as antiepileptics, allopurinol, sulphonamides, antibiotics, and non-steroidal anti-inflammatory drugs (Chen et al. 2010; Bluestein et al. 2021; Skowron et al. 2015; Fang et al. 2018).

The coexistence of different criteria is one of the reasons that hinders the diagnosis and assessment of DRESS (Kim and Koh 2014). Furthermore, the characterization of DILI associated with DRESS is controversial since there is no agreement about the criteria used to define liver injury in the context of immunoalergic reactions (Sanabria-Cabrera et al. 2019). Liver injury is usually defined as a mild elevation of transaminases (> 2 times the upper limit of normal [ULN]) (Kardaun et al. 2013), which is less stringent criteria than the thresholds proposed by an international DILI working group to exclude transaminase elevations of uncertain significance and an adaptative phenomena (Aithal et al. 2011). DILI associated with DRESS has gained interest in recent years, and several studies of Asian patients with this particular phenotype of DILI have been published recently (Huang et al. 2021; Devarbhavi et al. 2022).

In the setting of two prospective DILI cohorts with long-term follow-up, the Spanish DILI Registry and the Latin American DILI (LATINDILI) Network, we aimed to comprehensively characterize the causative drugs, clinical characteristics, laboratory features and outcomes of patients with DILI associated with DRESS (henceforth, DILI-DRESS).

## Methods

### Study population

Information from well-vetted idiosyncratic DILI cases included in the Spanish DILI Registry and the LATINDILI Network since their establishment until 2022 was collected. Details of these registries have been described elsewhere (Bessone et al. 2016; Stephens et al. 2021). A structured case report form was used to record pharmacological and clinical data, the description of skin lesions, blood test results, imaging findings to rule out other causes of liver damage, and the outcome of liver injury. The study protocols were approved by local ethics committees. All subjects gave informed written consent.

The biochemical criteria for DILI were those proposed by the Council for International Organizations of Medical Sciences (CIOMS) (Danan and Benichou 1993), later adapted to those set in 2011 (Aithal et al. 2011), i.e., serum alanine aminotransferase (ALT) elevation ≥ 5 × ULN, serum alkaline phosphatase (ALP) ≥ 2 × ULN, or the combination of ALT ≥ 3 × ULN with a simultaneous elevation of total bilirubin (TBL) > 2 × ULN. The pattern of liver injury was defined by the nR value, i.e., (ALT/ULN or aspartate aminotransferase [AST]/ULN, whichever highest ÷ ALP/ULN) (Robles-Diaz et al. 2014). Cases were classified as hepatocellular (nR ≥ 5), cholestatic (nR ≤ 2), or mixed (nR > 2 and < 5). Severity was graded into mild (TBL < 2 × ULN), moderate (TBL ≥ 2 × ULN), severe (TBL ≥ 2 × ULN, and either International Normalized Ratio [INR] ≥ 1.5, ascites and/or encephalopathy, or another organ failure due to DILI), and fatal or transplantation (liver-related death or liver transplantation) (Aithal et al. 2011). Time to DILI recognition (latency) was defined as the time from the start of drug intake to the onset of DILI. The number of patients meeting the nR-based Hy’s law criteria was calculated (Robles-Diaz et al. 2014). DILI cases were followed-up until liver injury resolution, i.e., all liver parameters below the upper limit of normal.

In all cases, other non-related drug causes of liver injury, such as viral hepatitis, biliary obstruction, alcoholism, autoimmune hepatitis, and according to the clinical context, metabolic disorders, ischemic hepatitis, septic shock, Epstein Barr, or cytomegalovirus infection, were excluded. A panel of experts evaluated the causal relationship between the suspected drug and liver damage. Case likelihood categorization was based on the categories of the Roussel Uclaf Causality Assessment Method (RUCAM) (Bessone et al. 2016; Stephens et al. 2021). Only cases that scored at least “possible” (≥ 3 points) were included.

DILI-DRESS cases were defined as those who had DILI and fulfilled at least three of the following criteria: acute skin rash, fever above 38 ºC, enlarged lymph nodes, internal organ involvement, or haematological abnormalities (lymphocytosis, lymphocytopenia, eosinophilia or thrombocytopenia) (Peyrière et al. 2006). Eosinophilia was defined, based on blood work at DILI recognition as serum eosinophils exceeding 4–6% of total leukocyte count depending on the normal range of individual hospitals, and lymphopenia as serum lymphocytes < 10%. The case ascertainment of DILI-DRESS was done based on the scoring system proposed by the RegiSCAR group (Peyrière et al. 2006). Only patients that scored at least “possible” were included. Stevens-Johnson syndrome (SJS), toxic epidermal necrolysis (TEN), and acute generalized exanthematous pustulosis (AGEP) cases were excluded.

For comparison purposes with the DILI-DRESS group, we defined a group of patients with DILI without DRESS (from now on, DILI). Cases with missing or incomplete information on any hypersensitivity features (fever, rash, eosinophilia, lymphopenia, and arthralgia) were excluded.

### Statistical analysis

Categorical data were expressed using frequency distributions, and differences were tested with the chi-square test or Fisher's exact test, as appropriate. For quantitative data, mean and standard deviation (SD), or median and interquartile range (IQR), were computed, and the Student's t-test or Mann–Whitney U test, as appropriate, were used to test differences between groups. A backward stepwise *logit* model was fitted to identify prognostic factors in DILI-DRESS cases. At each step, variables were chosen based on a p-value lower than a specified threshold of 0.05. All analyses were performed using STATA 17 (College Station, TX: StataCorp LLC), and a p-value lower than 0.05 was deemed statistically significant.

## Results

### Demographics, clinical characteristics, and outcome of DILI-DRESS and DILI cases

Out of 1,437 patients, we identified 53 DILI-DRESS cases (29 in the Spanish DILI Registry and 24 in the LATINDILI Network). Thus, the prevalence of DRESS (with confirmed DILI) in these registries was 3.7%. Two SJS/TEN/AGEP cases were excluded from this study. In addition, 881 DILI cases were included. No differences in demographic and clinical characteristics were found between DILI-DRESS cases from the Spanish DILI Registry and LATINDILI Network, except that Latin American patients were younger (mean age 54 years in the Spanish cases vs. 39 years in the Latin American cases; p = 0.006).

Demographics, clinical characteristics, and outcomes of DILI-DRESS and DILI cases were compared in Table 1. DILI-DRESS patients were younger than DILI (mean age 47 vs. 53 years, respectively; p = 0.042). However, when only Spanish cases were analysed, no differences in age were found (mean age 54 years in both groups; p = 0.927). Furthermore, there were significant differences in the pattern of liver damage (p = 0.018). Hepatocellular injury was predominant in DILI (63%), while 56% of DILI-DRESS patients presented with cholestatic/mixed damage. Indeed, the median elevation of ALP was significantly higher in DILI-DRESS cases (median 2.1 × ULN) than in DILI cases (median 1.6 × ULN; p = 0.003). Likewise, gamma-glutamyl transferase (GGT) levels were increased in DILI-DRESS cases compared to DILI (median 7.2 vs. 5.5 times ULN; p = 0.039).

**Table 1:** Comparison of demographics, clinical characteristics, laboratory parameters and outcome between DILI-DRESS and DILI cases

Characteristics	DILI-DRESS (n = 53)	DILI (n = 881)	p-value
Age (y), mean ± SD	47 ± 20	53 ± 18	0.042
Female, n (%)	28 (53)	475 (54)	0.878
BMI (kg/m^2^), mean ± SD	25 ± 4.0	26 ± 4.5	0.472
Diabetes mellitus, n (%)	3 (5.7)	93 (11)	0.352
Hypertension, n (%)	8 (17)	182 (24)	0.375
Dyslipidaemia, n (%)	6 (11)	75 (8.5)	0.451
Underlying hepatic disease, n (%)	3 (5.7)	55 (6.2)	1.000
History of drug allergy, n (%)	3 (6.7)	60 (8.2)	1.000
Type of liver injury, n (%)			0.018
Hepatocellular	23 (44)	513 (63)	
Cholestatic	14 (27)	173 (21)	
Mixed	15 (29)	133 (16)	
Duration of therapy (d), median (IQR)	31 (15–40)	30 (9–76)	0.331
Latency (d), median (IQR)	25 (15–36)	27 (10–62)	0.284
Number of concomitant medications, median (IQR)	1 (0–2)	1 (0–3)	0.143
Jaundice, n (%)	36 (68)	547 (63)	0.429
Hospitalization, n (%)	41 (79)	414 (50)	< 0.001
Fever, n (%)	29 (55)	78 (8.9)	< 0.001
Rash, n (%)	53 (100)	49 (5.6)	< 0.001
Arthralgia, n (%)	6 (14)	37 (4.2)	0.013
Lymphopenia, n (%)	17 (34)	127 (14)	< 0.001
Eosinophilia, n (%)	44 (85)	156 (18)	< 0.001
Positive autoantibody titres, n (%)	2 (5.1)	162 (21)	0.013
Laboratory parameters at DILI recognition (x ULN), median (IQR)			
Total bilirubin	4.0 (1.0–11)	4.2 (1.0–9.2)	0.799
Aspartate aminotransferase (AST)	5.1 (2.3–14)	6.1 (2.9–16)	0.481
Alanine aminotransferase (ALT)	8.7 (4.6–20)	9.1 (4.8–21)	0.694
Alkaline phosphatase (ALP)	2.1 (1.4–3.0)	1.6 (0.9–2.7)	0.003
Gamma-glutamyl transferase (GGT)	7.2 (4.0–11)	5.5 (2.4–11)	0.039
Creatinine (mg/dL)	0.8 (0.7–1.2)	0.9 (0.7–1.0)	0.900
Platelets (× 10^3^/mL)	198 (178–270)	223 (178–279)	0.358
Albumin (g/dL)	3.7 (3.1–4.1)	3.9 (3.4–4.3)	0.120
Neutrophil-to-lymphocyte ratio	1.8 (1.0–3.3)	2.2 (1.5–3.2)	0.196
Severity, n (%)			0.215
Mild	16 (30)	311 (35)	
Moderate	29 (55)	486 (55)	
Severe	7 (13)	52 (5.9)	
Fatal	1 (1.9)	29 (3.3)	
nR-based Hy’s law, n (%)	12 (24)	259 (33)	0.215
Outcome			
Time to resolution (d), median (IQR)	97 (60–180)	84 (47–153)	0.340
Liver-related death, n (%)	1 (1.9)	16 (1.8)	1.000
Liver transplantation, n (%)	0 (0)	13 (1.5)	1.000
Death due to other causes^a^, n (%)	3 (5.7)	9 (1.0)	0.026

*ALP* alkaline phosphatase; *ALT* alanine aminotransferase; *AST* aspartate aminotransferase; *BMI* body mass index; *d* days; *DILI* drug-induced liver injury; *DRESS* Drug Reaction with Eosinophilia and Systemic Symptoms; *IQR* interquartile range (25–75%); SD: standard deviation; *ULN* upper limit of normal; *y* years

^a^During time of follow-up

All DILI-DRESS cases had rash, and 85% presented with eosinophilia. Moreover, 55% of these patients had fever, 34% had lymphopenia, and only 14% suffered from arthralgia. When compared to DILI, DILI-DRESS cases had a lower prevalence of positive autoantibody titres (21% and 5.1%, respectively; p = 0.013), and the hospitalization rate was higher (50% vs. 79%, respectively; p < 0.001).

Eleven DILI-DRESS patients underwent liver biopsy. Histological findings showed cholestasis with hepatitis (n = 4), zonal necrosis (n = 3), chronic hepatitis (n = 2, active in one of them), steatohepatitis (n = 1), and unspecific changes in the liver (n = 1).

Even though the damage was moderate in most patients in both groups (55%), those in the DILI-DRESS group developed a severe liver injury more frequently than DILI cases (13% vs. 5.9%, respectively), albeit these differences did not reach statistical significance. Of note, 13 out of 53 DILI-DRESS cases (25%) were treated with corticosteroids. Furthermore, there were no differences in liver-related death. Only one DILI-DRESS patient, due to anti-tuberculosis (TB) drugs died (after liver transplantation). Conversely, death due to non-liver-related causes was higher among the DILI-DRESS patients (5.7%; n = 3), compared to DILI (1.0%; n = 9) (p = 0.026). Information of the 53 DILI-DRESS patients is further detailed in Table 2.

**Table 2:** Demographics, clinical and biochemical characteristics of the 53 patients with DILI-DRESS

Sex/Age (y)	Indication	Clinical presentation	Underlying disease	Drug	Doses (mg/d)	Concomitant medication	Latency (d)	TB (X ULN)	ALT (X ULN)	AST (X ULN)	ALP (X ULN)	INR	Pattern of liver injury (nR-ratio). Histological findings	Type of skin injury	Severity	Outcome (d)
M/69	Dyspepsia	Rash, eosinophilia, jaundice, hospitalization	None	Omeprazole	20	None	10	18	24	18	1.3	1.4	Hepatocellular (17.9). Cholestasis with hepatitis	Rash	Moderate	Recovery (604)
M/33	Ulcerative colitis	Rash, fever, hospitalization	None	Sulfasalazine	2000	Clindamycin, ciprofloxacin	28	0.9	5.7	2.6	1.7	NA	Mixed (3.3)	Erythema (generalized)	Mild	Recovery (364)
F/53	Diabetes mellitus	Rash, fever, jaundice, hospitalization	Diabetes	Metformin; gliclazide	850; 60	Paroxetine, lorazepam, levothyroxine	43	NA	9.4	4.4	2.8	NA	Mixed (3.4)	Pruritus	Moderate	Lost to follow-up (19)
M/70	Post-coronary stenting prophylaxis	Rash, eosinophilia, jaundice	Hypertension	Ticlopidine	500	Atenolol	21	8.2	3.0	1.5	2.9	1.6	Cholestatic (1.0)	Rash, pruritus	Moderate	Lost to follow-up (180)
F/76	Atrial fibrillation	Rash, fever, lymphopenia, jaundice, hospitalization	Renal diseases, cardiovascular disease, hypothyroidism, metabolic disorders	Propafenone	450	Levothyroxine, allopurinol, spironolactone, furosemide, amiodarone, warfarin	60	10	3.3	3.1	4.7	NA	Cholestatic (0.7)	Rash	Severe	Lost to follow-up (322)
F/60	Hyperlipidemia, osteoporosis	Rash, fever, lymphopenia, eosinophilia, jaundice, hospitalization	Neoplastic diseases	Fenofibrate; raloxifene	250; 60	None	14	6.2	6.7	5.1	1.7	1.1	Mixed (4.0). Zonal necrosis	Exanthem (upper body and limbs)	Moderate	Lost to follow-up (544)
F/56	Menopause; disc herniation	Rash, eosinophilia, jaundice, hospitalization	Asthma	Progesterone and estrogen; diclofenac	1.1; 150	Betamethasone, paracetamol, ferrous glycine sulfate, progesterone	547	12	40	51	3.5	1.6	Hepatocellular (14.4). Active chronic hepatitis	Rash (upper body and upper limbs)	Moderate	Recovery (134)
F/27	Obesity	Rash, fever, lymphopenia, jaundice, hospitalization	None	Tiratricol	1.4	Desogestrel and estrogen, mazindol, potassium chlorazepate, *Cassia angustifolia*	35	8.2	8.1	7.4	2.6	1.1	Mixed (3.2). Cholestasis with hepatitis	Exanthem (upper limbs)	Moderate	Recovery (187)
M/16	Acne	Rash, fever, eosinophilia, jaundice, hospitalization	None	Minocycline	200	None	42	2.3	15	31	9.5	NA	Mixed (3.2)	Erythema (generalized), pruritus	Moderate	Recovery (63)
M/65	Respiratory infection; neuralgia	Rash, fever, lymphopenia, eosinophilia, jaundice	None	Amoxicillin-clavulanate; carbamazepine	1875; 600	Clarithromycin	4	21	5.9	16	9.7	NA	Cholestatic (1.6). Cholestasis with hepatitis	Exanthem, pruritus	Moderate	Recovery (1,595)
M/82	Respiratory infection	Rash, eosinophilia, hospitalization	Dyslipidemia	Amoxicillin-clavulanate	1875	Ampicillin, carbocisteine	2	1.9	5.6	3.1	1.6	1.0	Mixed (3.5)	Pruritus (generalized)	Mild	Lost to follow-up (79)
M/60	NA	Rash, lymphopenia, eosinophilia, jaundice, hospitalization	Chronic obstructive pulmonary disease	Amoxicillin-clavulanate	1875	Clarithromycin, deflazacort, theophylline, salmeterol, tiotropium bromide	32	12	3.2	1.9	3.2	1.0	Cholestatic (1.0). Unspecific changes	Rash, pruritus, petechias (upper body and upper limbs)	Moderate	Non-liver related death
M/25	Pneumonia	Rash, eosinophilia, hospitalization	None	Amoxicillin-clavulanate; piperacillin	3600; 4500	Metamizole sodium, paracetamol, haloperidol, phenytoin, ibuprofen, amikacin, pantoprazole, domperidone, lactulose, vancomycin, dalteparin, midazolam	22	0.2	20	45	2.6	2.2	Hepatocellular (17.3)	Rash, pruritus	Mild	Recovery (56)
M/67	Skin wound infection	Rash, fever, lymphopenia, eosinophilia, arthralgia, jaundice, hospitalization	Diabetes, hypertension	Amoxicillin-clavulanate	1000	None	9	12	4.6	1.9	1.5	0.7	Mixed (3.1)	Erythema (generalized), pruritus	Moderate	Lost to follow-up (35)
F/48	Rhinosinusitis	Rash, eosinophilia, jaundice, hospitalization	None	Amoxicillin-clavulanate	1875	None	34	5.3	8.7	6.8	1.0	1.0	Hepatocellular (8.9)	Rash	Moderate	Recovery (97)
F/17	Ankylosing spondylitis	Rash, fever, eosinophilia, arthralgia, hospitalization	None	Sulfametoxydiazine	1500	Naproxen, paracetamol, esomeprazole	16	NA	2.8	1.8	3.0	1.5	Cholestatic (0.9)	Pruritus, micropapules (generalized)	Mild	Recovery (43)
M/61	Prostate infection	Rash, eosinophilia, jaundice	None	Sulfamethoxazole and trimethoprim	840	None	33	4.0	14	6.5	2.5	0.9	Hepatocellular (5.3)	Pruritus (upper limb, back)	Moderate	Recovery (105)
M/50	Prostate infection	Rash, eosinophilia, jaundice	None	Sulfamethoxazole and trimethoprim	1920	None	25	15	4.7	3.8	1.5	1.0	Mixed (3.1)	Rash	Moderate	Recovery (288)
M/51	Acute bronchitis	Rash, lymphopenia, eosinophilia, jaundice, hospitalization	None	Ciprofloxacin	400	None	7	1.8	8.8	7.2	2.1	1.0	Mixed (4.2)	Rash	Mild	Lost to follow-up (20)
M/68	Respiratory infection	Rash, lymphopenia, eosinophilia, jaundice, hospitalization	Chronic obstructive pulmonary disease, cardiovascular disease, peripheral vascular disease	Trovafloxacin	200	Pentoxifylline	15	9.8	64	32	2.1	1.1	Hepatocellular (30.2). Cholestasis with hepatitis	Pruritus (generalized)	Moderate	Recovery (81)
M/77	Respiratory infection	Rash, fever, lymphopenia, jaundice, hospitalization	Chronic obstructive pulmonary disease, cardiovascular disease, dyslipidemia	Moxifloxacin	400	Simvastatin, carbocisteine	2	7.2	5.7	1.8	1.2	NA	Mixed (4.9)	Pruritus with papules (generalized)	Moderate	Lost to follow-up (27)
M/27	Respiratory infection; pain	Rash, eosinophilia, jaundice, hospitalization	None	Isoniazid; paracetamol	300; 3000	Acetylsalicylic acid, ibuprofen, erythromycin, paracetamol	21	17	57	35	0.5	2.5	Hepatocellular (113.4). Zonal necrosis	Rash	Severe	Recovery (150)
M/64	Tuberculosis	Rash, eosinophilia, jaundice, hospitalization	Hypertension, peripheral vascular disease, peptic ulcer disease, upper gastrointestinal bleeding, hepatic disease	Pyrazinamide	1500	Ciprofloxacin, isoniazid, rifampicin	6	11	0.5	2.3	0.7	2.5	Hepatocellular (3.0)	Rash (generalized)	Severe	Non-liver related death
M/61	Extrapulmonary tuberculosis	Rash, fever, arthralgia, jaundice, hospitalization	None	RIP + INH + PIZ	1025	Ethambutol	4	1.1	9.0	3.5	1.2	NA	Hepatocellular (7.6)	Rash	Severe	Lost to follow-up (50)
F/14	Tuberculosis	Rash, eosinophilia	Hepatic disease	RIP + INH + PIZ	900	Streptomycin	17	1.0	14	7.8	2.5	1.4	Hepatocellular (5.7). Steatohepatitis	Rash	Mild	Lost to follow-up (50)
F/18	Tuberculosis	Rash, fever, lymphopenia, hospitalization	None	RIP + INH + PIZ	2500	None	33	11	61	51	1.9	NA	Hepatocellular (32.8)	Rash	Fatal	Liver-related death
F/34	Tuberculosis	Rash, fever, lymphopenia, jaundice, hospitalization	None	RIP + INH + PIZ	1875	None	25	0.5	1.9	1.6	3.0	NA	Cholestatic (0.6)	Rash	Mild	Recovery (233)
F/37	Tuberculosis	Rash, fever, eosinophilia, jaundice	None	RIP + INH + PIZ	3,600	None	24	11	41	38	1.4	NA	Hepatocellular (29.7)	Exanthem (generalized)	Severe	Non-liver related death
F/42	HIV infection	Rash, fever, eosinophilia, jaundice, hospitalization	Hepatic disease	Nevirapine	400	Lamivudine, zidovudine	7	7.8	200	168	1.6	3.4	Hepatocellular (127.1)	Rash (upper body)	Severe	Recovery (114)
F/56	Melanoma	Rash, eosinophilia	None	Nivolumab; ipilimumab	3; 1 (cycles)	None	72	1.6	13	11	1.1	NA	Hepatocellular (12.2)	Rash	Moderate	Lost to follow-up (98)
M/38	Low back pain	Rash, eosinophilia, jaundice, hospitalization	None	Diclofenac	120	Ebastine, codeine	37	1.2	46	21	1.8	NA	Hepatocellular (26.3). Chronic hepatitis	Rash	Mild	Recovery (903)
F/81	Skin wound infection	Rash, lymphopenia, eosinophilia, jaundice, hospitalization	Hypertension	Amoxicillin-clavulanate; meloxicam	NA	Quinapril, amiloride	7	13	2.4	1.7	2.0	NA	Cholestatic (1.2)	Pruritus	Moderate	Recovery (180)
F/68	Hyperuricemia	Rash, fever, eosinophilia, hospitalization	Dyslipidemia, hypertension, metabolic disorders	Allopurinol	300	Ebastine	30	0.7	21	13	NA	NA	Hepatocellular	Exanthem (face, upper body)	Moderate	Recovery (65)
M/70	NA	Rash, fever, eosinophilia	Dyslipidemia, hypertension	Allopurinol	300	Fenofibrate, telmisartan	31	0.9	4.8	1.2	1.4	0.8	Mixed (3.4)	Rash	Mild	Lost to follow-up (8)
M/56	Hyperuricemia	Rash, fever, lymphopenia, eosinophilia, hospitalization	Diabetes, hypertension	Allopurinol	300	Atorvastatin, valsartan, methyldopa, carvedilol	40	0.7	8.4	3.2	1.6	1.3	Hepatocellular (5.3)	Petechia (generalized)	Mild	Recovery (51)
M/46	Hyperuricemia; hypercholesterolemia	Rash, fever, eosinophilia, jaundice, hospitalization	None	Allopurinol; atorvastatin	300; 10	None	38	4.0	7.2	8.3	3.9	1.2	Mixed (2.1)	Rash	Moderate	Recovery (92)
F/60	Epilepsy	Rash, fever, eosinophilia, jaundice, hospitalization	None	Phenytoin; oxcarbazepine	100; 300	Levetiracetam, ranitidine	19	4.4	26	47	1.7	1.3	Hepatocellular (27.5)	Rash, exanthem, pruritus	Moderate	Lost to follow-up (12)
M/28	Neurosurgical prophylaxis; skin wound infection	Rash, fever, lymphopenia, hospitalization	None	Phenytoin; clindamycin	300; 600	Ceftriaxone	16	0.4	10	4.8	1.1	NA	Hepatocellular (9.7)	Pharmacodermia	Mild	Recovery (68)
F/14	Epilepsy	Rash, lymphopenia, eosinophilia	None	Carbamazepine	200	None	21	3.8	17	8.6	3.9	0.8	Mixed (4.3)	Rash, pruritus	Moderate	Recovery (29)
F/63	Neuralgia	Rash, eosinophilia, jaundice, hospitalization	None	Carbamazepine	600	None	21	3.5	19	13	4.9	NA	Mixed (3.8)	Toxicodermia	Moderate	Lost to follow-up (30)
F/72	Neuralgia	Rash, eosinophilia, jaundice	Hypothyroidism, hypertension, meningioma	Carbamazepine	600	Levothyroxine, ramipril	46	10	1.9	2.1	13	0.8	Cholestatic (0.2)	Rash	Moderate	Recovery (111)
F/42	Schizophrenia	Rash, fever, eosinophilia, jaundice, hospitalization	None	Carbamazepine	400	Risperidone	36	3.2	2.2	2.3	6.5	NA	Cholestatic (0.4)	Rash	Moderate	Recovery (134)
F/48	Muscle pain	Rash, fever, eosinophilia, arthralgia, jaundice, hospitalization	None	Carbamazepine	100	Acetylsalicylic acid	31	4.7	3.3	1.2	6.6	NA	Cholestatic (0.5)	Rash, pruritus (upper body, lower limbs)	Moderate	Recovery (42)
F/19	Adjustment disorder	Rash, eosinophilia, jaundice, hospitalization	None	Carbamazepine	200	Azithromycin, penicillin	42	NA	6.1	2.9	NA	NA	NA	Rash, pruritus, exanthem (face)	Moderate	Recovery (60)
M/27	Seizures	Rash, eosinophilia, jaundice	None	Carbamazepine	600	None	34	12	20	8.0	1.7	1.0	Hepatocellular (12.1)	Rash	Moderate	Recovery (479)
F/50	Depression	Rash, fever, lymphopenia, eosinophilia, jaundice, hospitalization	None	Valproic acid	1000	Paroxetine, alprazolam	15	3.7	11	8.7	3.0	NA	Mixed (3.8)	Exanthem, pruritus	Moderate	Lost to follow-up (70)
M/16	Sinusitis	Rash, eosinophilia, arthralgia, hospitalization	None	Lamotrigine	25	Prednisolone	3	0.6	15	4.4	2.6	NA	Hepatocellular (5.6)	Rash	Mild	Lost to follow-up (36)
F/40	Depression	Rash, fever, eosinophilia, hospitalization	Metabolic disorders	Lamotrigine; quetiapine	150; 12.5	None	40	0.4	2.0	0.4	2.9	2.3	Cholestatic (0.7)	Rash (generalized)	Mild	Recovery (60)
M/51	Depression	Rash, fever, eosinophilia, jaundice, hospitalization	None	Lamotrigine	100	None	48	14	50	31	1.2	3.9	Hepatocellular (42.3)	Rash	Severe	Lost to follow-up (9)
F/19	Depression	Rash, fever, eosinophilia, jaundice, hospitalization	None	Lamotrigine	75	None	39	3.4	27	14	0.4	NA	Hepatocellular (75.1)	Pharmacodermia	Moderate	Recovery (62)
F/18	Epilepsy	Rash, fever, lymphopenia, eosinophilia, hospitalization	None	Levetiracetam	1000	None	33	0.7	3.5	1.4	3.8	1.3	Cholestatic (0.9). Zonal necrosis	Rash (generalized)	Mild	Lost to follow-up (104)
F/32	Bipolar disorder	Rash, fever, eosinophilia, arthralgia, hospitalization	None	Levomepromazine	6	Valproic acid, lorazepam, oxcarbazepine	22	0.9	5.4	4.2	2.8	NA	Cholestatic (2.0)	Rash	Mild	Lost to follow-up (43)
F/51	Schizophrenia	Rash, eosinophilia	Hypertension	Olanzapine	30	Potassium clorazepate, clomipramine	20	0.1	2.7	1.7	2.2	0.9	Cholestatic (1.2)	Rash (generalized), pruritus	Mild	Recovery (19)

*DILI* drug-induced liver injury; *DRESS* Drug Reaction with Eosinophilia and Systemic Symptoms; *M* male; *F* female; *y* years; *d* days; *TB* total bilirubin; *ALT* alanine transaminase; *AST* aspartate transaminase; *ALP* alkaline phosphatase; *ULN* upper limit of normal; *INR* International Normalized Ratio; *RIP* + *INH* + *PIZ* combination of rifampicin, isoniazid and pyrazinamide; *HIV* Human immunodeficiency virus; *NA* not available

### Prognostic factors in DILI-DRESS cases

DILI-DRESS patients who developed a severe-fatal injury had predominantly a hepatocellular injury (88%) and marked elevations of serum transaminases and TBL levels at DILI recognition compared to those with mild-to-moderate liver damage. In addition, 63% of cases with a more severe injury presented with eosinophilia, compared to 89% of patients with milder damage.

An exploratory backward stepwise regression analysis was performed to identify factors associated with the development of a worse outcome in DILI-DRESS cases out of the following variables (p-value < 0.1 in univariate analysis): type of liver injury (hepatocellular vs. cholestatic/mixed), eosinophilia, ALT, AST, ALP, TBL, and nR-based Hy's law. Of these, higher TBL levels at DILI recognition (odds ratio [OR] 1.23; 95% confidence interval [CI] 1.04–1.45) and absence of eosinophilia (OR 8.77; 95% CI 1.11–69.20) were found as prognostic factors of worse outcome in DILI-DRESS patients.

### Causative agents

The most common agents implicated in DILI-DRESS were carbamazepine (13%), anti- TB medications (isoniazid, rifampicin, and pyrazinamide, either alone or in combination, 13%), amoxicillin-clavulanate (11%), and allopurinol and lamotrigine (7.6% each). On the other hand, amoxicillin-clavulanate (15%), anti-TB (6.6%), ibuprofen (3.5%), and diclofenac (3.1%) were the most frequent causative drugs in DILI cases. Notably, among all cases of carbamazepine-induced liver injury in the two registries (n = 15), 47% of them were DILI-DRESS, as well as four out of six cases due to lamotrigine (67%). However, in cases due to other antiepileptics such as valproic acid or phenytoin (n = 9 each), few of them developed DILI-DRESS (11% and 22%, respectively). Conversely, among the six cases of DILI due to allopurinol, 67% presented with a DILI-DRESS phenotype.

According to the Anatomic Therapeutic Classification groups, anti-infectives for systemic use were the most common drugs in both groups (40% in DILI-DRESS and 34% in DILI). Furthermore, drugs for the nervous system were overrepresented in DILI-DRESS compared to DILI (32% vs. 8.6%, respectively). In contrast, drugs for the cardiovascular system and antineoplastic and immunomodulating agents were more common among DILI patients (Online Resource 1).

An ancillary analysis was performed to compare the clinical characteristics of DILI-DRESS patients according to the most frequent culprit drugs and drug classes, i.e., antiepileptic drugs (carbamazepine, lamotrigine, phenytoin, valproic acid and levetiracetam), anti-TB drugs, amoxicillin-clavulanate, and allopurinol (Table 3). Young women were more prone to present DILI-DRESS caused by antiepileptic or anti-TB medications. DILI-DRESS was induced by amoxicillin-clavulanate after a shorter duration of therapy. Hepatocellular injury was distinctive in DILI-DRESS caused by anti-TB drugs, while the cholestatic/mixed pattern of liver injury was the predominant damage caused by the other drugs. Moreover, it is worth noting that eosinophilia was less prevalent in those DILI-DRESS patients who had taken anti-TB medications and had a poorer outcome.

**Table 3:** Characteristics of DILI-DRESS cases caused by the most frequent culprit drug and drug classes

Characteristics	Antiepileptic drugs (n = 15)	Anti-tuberculosis drugs (n = 7)	Amoxicillin-clavulanate (n = 6)	Allopurinol (n = 4)
Age (y), mean ± SD	38 ± 19	36 ± 20	58 ± 19	60 ± 11
Female, %	73	57	17	25
Type of liver injury, %				
Hepatocellular	43	86	33	50
Cholestatic	36	14	33	0
Mixed	21	0	33	50
Duration of therapy (d), median (IQR)	40 (30–45)	32 (21–34)	7 (4–10)	35 (27–39)
Latency (d), median (IQR)	33 (19–40)	21 (6–25)	16 (4–32)	35 (31–39)
Number of concomitant medications, median (IQR)	1 (0–2)	1 (0–3)	2 (0–5)	2 (1–3)
Eosinophilia, %	93	57	100	100
Laboratory parameters at DILI recognition (x ULN), median (IQR)				
Total bilirubin	3.6 (0.7–4.7)	11 (1.0–11)	8.4 (1.9–12)	0.8 (0.7–2.5)
Aspartate aminotransferase (AST)	4.8 (2.1–13)	7.8 (2.3–38)	4.9 (1.9–16)	5.7 (2.2–11)
Alanine aminotransferase (ALT)	11 (3.3–20)	14 (1.9–57)	5.7 (4.6–8.7)	7.8 (6.0–15)
Alkaline phosphatase (ALP)	2.9 (1.7–4.9)	1.4 (0.7–2.5)	2.1 (1.4–3.2)	1.6 (1.4–3.9)
Severity, %				
Mild	27	29	33	50
Moderate	67	0	67	50
Severe	6.7	57	0	0
Fatal	0	14	0	0
nR-based Hy’s law, %	23	43	17	0
Outcome				
Time to resolution (d), median (IQR)	62 (60–111)	192 (150–233)	97 (56–1,595)	65 (51–92)
Liver-related death, n (%)	0 (0)	1 (14)	0 (0)	0 (0)
Liver transplantation, n (%)	0 (0)	0 (0)	0 (0)	0 (0)
Death due to other causes, n (%)	0 (0)	2^a^ (29)	1 (17)	0 (0)

*AST* aspartate aminotransferase; *ALT* alanine aminotransferase; *ALP* alkaline phosphatase; *d* days; *DILI* drug-induced liver injury; *DRESS* Drug Reaction with Eosinophilia and Systemic Symptoms; *IQR* interquartile range; *SD* standard deviation; *ULN* upper limit of normal

^a^DILI played a contributory role in the death of one case

## Discussion

In the present study, including cases of two prospective DILI registries from Western countries, we have comprehensively characterized a cohort of DILI-DRESS cases. Compared to DILI, DILI-DRESS presented more frequently with a cholestatic or mixed injury. Furthermore, these cases were more frequently hospitalized and tended to course with a more severe injury, albeit their prognosis was comparable to DILI cases. Causative agents responsible for DILI-DRESS showed a distinctive pattern, with antiepileptics as the leading drug class.

A summary of the clinical characteristics and outcomes of DILI-DRESS patients in prospective and retrospective studies published from Asian countries is shown in Table 4. The prevalence of DILI-DRESS in this study (3.7%) is similar to other long-term population-based studies (5.3%) (Huang et al. 2021), but significantly lower compared to a recent single-centre Indian study (19%) (Devarbhavi et al. 2022). Geographic, ethnic, and demographic differences and divergences in drug prescription patterns could explain these discrepancies in DILI-DRESS incidence. For instance, sulphonamides, mainly dapsone, rarely used in Western countries, was the 2nd drug class involved in DILI-DRESS in one of these studies (Devarbhavi et al. 2022). Furthermore, the increased mortality rates observed in these studies could be due to several factors, such a higher prevalence of pre-existing hepatic diseases, comorbidities, or a more frequent involvement of extrahepatic organs, which might have determined the poorer outcome (Chalasani et al. 2015; Ghabril et al. 2019).

**Table 4:** Demographics, clinical information, and outcome of DILI-DRESS cases in prospective and retrospective studies

	Lee et al. 2013 n = 136	Lin et al. 2015 n = 72	Huang et al. 2021 n = 1,415	Devarbhavi et al. 2022 n = 943
Years	2008–2011	2000–2013	2011–2020	1998–2021
Type of study	Retrospective	Retrospective	Prospective	Prospective
Study group	DRESS with liver dysfunction	DRESS with liver injury	DILI with DRESS	DILI with DRESS
Total DRESS cases, n	33	72	74	179
DRESS cases with liver injury, n (%)	23 (70)	62 (86)	74 (100)	179 (100)
Diagnostic criteria for DRESS	RegiSCAR	RegiSCAR	RegiSCAR	RegiSCAR
Causality assessment	WHO-UMC	ND	RUCAM	RUCAM
Acute skin eruption, %	ND	ND	ND	ND
Fever, %	ND	79	ND	ND
Eosinophilia, %	74	ND	91	60
Lymphopenia, %	30	74	ND	ND
Organ involvement other than liver, %	ND	ND	Kidney (34) Lung (27) Heart (11)	Bone marrow failure (10) Acute kidney injury (7.2 Thyroiditis (2.8) Carditis (2.2)
DILI criteria	AST or ALT > 40 IU/L, ALP > 120 IU/L, TBL > 1.2 mg/dL, or PT > 1.3 INR	ALT, AST, ALP, or direct bilirubin > 2 × ULN	ALT/AST > 5 × ULN, ALP > 2 × ULN, or any elevation in ALT, AST, or ALP and TBL > 2.5 mg/dL	ALT ≥ 5 × ULN, ALP ≥ 2 × ULN, or ALT ≥ 3 × ULN and TBL ≥ 2 × ULN
Age (years), mean ± SD	56 ± 14	49 (6–88)^b^	53 ± 18	34 ± 14
Female, %	48	53	43	54
Type of liver injury, %	ND	Hepatocellular (19) Cholestatic (37) Mixed (27)	Hepatocellular (31) Cholestatic (39) Mixed (30)	Hepatocellular (37) Cholestatic (41) Mixed (22)
Jaundice, %	ND	ND	43	53
Culprit drugs, %	Antibiotics (57) NSAID, allopurinol (13) Anticonvulsants (9) Herbal medicine (4)	Antiepileptic drugs (31) Allopurinol (24) Sulphonamides and sulfones (21) TMP-SMX, beta-lactam antibiotics (4.8)	Carbamazepine (27) TMP-SMX (26) Phenytoin (20) Allopurinol (15) Diclofenac (11) Anti-tuberculosis drugs (1.4)	Antiepileptic drugs (36) Sulfonamides (29) Anti-tuberculosis drugs (14) Non-mycobacterial antibiotics (10) Anti-retroviral drugs (8)
*Laboratory parameters at recognition, median (IQR)*				
Total bilirubin, mg/dL	1.1 (IQR 0.8–13.3)^a^	ND	3.4 ± 4.1^c^	5.5 ± 6.4^‡^
Aspartate aminotransferase (AST), IU/L	207 (90–766)^a^	ND	ND	266 (152–480)
Alanine aminotransferase (ALT), IU/L	186 (114–458)^a^	ND	507 ± 578^c^	328 (192–570)
Alkaline phosphatase (ALP), IU/L	147 (116–338)^a^	ND	312 ± 185^c^	252 (158–424)
All-cause mortality, n (%)	4 (17)	ND	10 (14)	14 (7.8)

*ALP* alkaline phosphatase; *ALT* alanine aminotransferase; *AST* aspartate aminotransferase; *DILI* drug-induced liver injury; *DRESS* Drug Reaction with Eosinophilia and Systemic Symptoms; *INR* International Normalized Ratio; *IQR* interquartile range (25–75%); *ND* no data available; *NSAID* non-steroidal anti-inflammatory drugs; *PT* prothrombin time; *RUCAM* Roussel Uclaf Causality Assessment Method; *SD* standard deviation; *TMP-SMX* trimethoprim-sulfamethoxazole; *ULN* upper limit of normal; *WHO-UMC* World Health Organization – Uppsala Monitoring Center

^a^Peak values

^b^Median age and range

^c^Mean and standard deviation

In the present study cholestatic-mixed injury was reported as the most common liver injury pattern in patients with DILI-DRESS. This finding is consistent with other studies that reported a significantly higher frequency of cholestatic injury in DILI-DRESS cases (Lin et al. 2015; Huang et al. 2021; Devarbhavi et al. 2022).

Antiepileptics were the most common drug class in our study. These drugs are metabolized by the cytochrome P450 to arene oxide metabolites, which are usually detoxified by the epoxide hydrolase or glutathione transferase to inactive metabolites (Spielberg et al. 1981). However, some investigations have reported that patients with DRESS caused by antiepileptics had a reduced detoxification capability due to defects in the epoxide hydrolase enzyme, resulting in the accumulation of reactive metabolites that may act as haptens that trigger an immune response (Shear and Spielberg 1988; Chung et al. 2014). This circumstance might explain the overrepresented incidence due to antiepileptic drugs despite DILI-DRESS and DILI share common pathogenic pathways (Cueto-Sanchez et al. 2021; Stirton et al. 2022).

One-third of DILI-DRESS caused by antiepileptics or anti-TB medications fulfilled the nR-based Hy's law. However, none of those cases caused by antiepileptics evolved into fatal outcomes. Conversely, one of the two cases treated with anti-TB died of acute liver failure. These findings underscore that Hy’s Law prognostic performance may vary with individual drugs (Stephens et al. 2021). Interestingly, DILI-DRESS cases due to anti-TB, who exhibited the highest mortality rate, had a lower prevalence of eosinophilia. Indeed, in our exploratory analysis, higher TBL levels and lack of eosinophilia were associated with a worse outcome in DILI-DRESS patients, underlining the role of eosinophilia in the prognosis of DILI-DRESS. Consistently, prior investigations have described an association between eosinophilia and a more favourable outcome of liver injury in DILI (Björnsson et al. 2007; Pachkoria et al. 2008). These findings indicate that liver-related mortality in DILI-DRESS depends on the culprit agent.

The main strength of this study is the large sample of patients recruited in these two prospective DILI registries with a standardized methodology. In addition, only DILI-DRESS cases that met international criteria were included, ensuring the internal validity of our findings. However, some limitations should be acknowledged. Information about skin lesions was reported in the standardized case report form, but no skin biopsies were available. Nonetheless, only cases that fulfilled the RegiSCAR criteria and were considered in the case ascertainment as possible or probable (Peyrière et al. 2006) were included as DILI-DRESS. Moreover, data from some biochemical parameters to further characterize these DILI-DRESS patients were not available, such as metabolomic profiles. Therefore, future studies should be planned to investigate if severity in DILI-DRESS patients is associated with a distinctive bile acid profile as occurs in DILI cases (Ma et al. 2019; Xie et al. 2021).

In conclusion, in this well-characterized cohort from two long-term prospective DILI registries, DILI-DRESS cases presented more frequently with a cholestatic/mixed injury pattern. They exhibited greater severity but similar rates of liver-related death and liver transplantation than DILI. Antiepileptic drug class was found as a distinctive causative drug group in DILI-DRESS. Lamotrigine and allopurinol have a greater chance of presenting with this phenotype. Exploratory analyses identified higher TBL levels and lack of eosinophilia as prognostic factors of poor outcomes. These findings represent a step forward to deepen the understanding of the distinctive clinical features and prognosis of DILI associated with DRESS.

### Supplementary Information

Below is the link to the electronic supplementary material.

Supplementary file1 (PDF 198 KB)

## Data Availability

The datasets generated and/or analysed during the current study are available from the corresponding author on reasonable request.
