# Supplementary material for: Characterization of drug-induced liver injury associated with drug reaction with eosinophilia and systemic symptoms in two prospective DILI registries
Source: Arch Toxicol. 2023 Dec 5;98(1):303–25. doi: 10.1007/s00204-023-03630-0 (PMC10761448; doi:10.1007/s00204-023-03630-0)
Supplement: Supplementary file 1 — Supplementary file1 (PDF 198 KB) [file 204_2023_3630_MOESM1_ESM.pdf]

Characterization of drug-induced liver injury associated with drug reaction with eosinophilia and systemic symptoms in two prospective DILI registries

Journal: *Archives of Toxicology*

Inmaculada Medina-Cáliz[1†], Judith Sanabria-Cabrera[1,2,3†], Marina Villanueva-Paz[1,2†], Lauryna Aukštikalnė[4], Camilla Stephens[1,2], Mercedes Robles-Díaz[1,2], José M. Pinazo-Bandera[1,2], Miren García-Cortes[1,2], Isabel Conde[2,5], German Soriano[2,6], Fernando Bessone[7], Nelia Hernandez[8], Vinicius Nunes[9], Raymundo Paraná[9], M. Isabel Lucena[1,2,3*], Raúl J. Andrade[1,2,3‡], Hao Niu[1,2,3‡], Ismael Alvarez-Alvarez[1,2,3‡]

[1] Servicios de Aparato Digestivo y Farmacología Clínica, Hospital Universitario Virgen de la Victoria, Instituto de Investigación Biomédica de Málaga y Plataforma en Nanomedicina-IBIMA Plataforma BIONAND, Universidad de Málaga, Málaga, Spain

[2] Centro de Investigación Biomédica en Red Enfermedades Hepáticas y Digestivas (CIBERehd), Madrid, Spain

[3] Plataforma de Investigación Clínica y Ensayos Clínicos UICEC-IBIMA, Plataforma ISCIII de Investigación Clínica, Madrid, Spain

[4] Lithuanian University of Health Sciences, Institute of Physiology and Pharmacology, Kaunas, Lithuania

[5] Unidad de Hepatología, Servicio de Aparato Digestivo, Hospital Universitari i Politècnic La Fe, Valencia, Spain

[6] Servicio de Gastroenterología, Hospital de la Santa Creu i Sant Pau, Universitat Autònoma de Barcelona, Barcelona, Spain

[7] Hospital Provincial del Centenario, Rosario, Argentina

[8] Hospital de Clínicas, Montevideo, Uruguay

[9] Hospital Universitário Prof. Edgard Santos-UFBA, Salvador, Brazil

[†] I.M.-C., J.S.-C. and M.V.-P. equally contributed as first authors.

[‡] R.J.A., H.N. and I.A.-A. equally contributed as senior authors.

[*] **Correspondence:**

M. Isabel Lucena

E-mail: lucena@uma.es

Supplemental Table 1. Main Anatomic Therapeutic Classification (ATC) groups in DILI-DRESS and DILI cases.

ATC group and subgroup	DILI-DRESS (n=53) n (%)	DILI (n=881) n (%)
**A (Alimentary tract and metabolism)**	**3 (5.7)**	**36 (4.1)**
A02 (Drugs for acid related disorders)	1 (1.9)	21 (2.4)
A03 (Drugs for functional gastrointestinal disorders)	-	3 (0.3)
A07 (Antidiarrheals, intestinal anti-inflammatory / antiinfective agents)	1 (1.9)	4 (0.5)
A08 (Antiobesity preparations, excluding diet products)	-	3 (0.3)
A10 (Drugs used in diabetes)	1 (1.9)	4 (0.5)
A11 (Vitamins)	-	1 (0.1)
**B (Blood and blood forming organs)**	**1 (1.9)**	**13 (1.5)**
B01 (Antithrombotic agents)	1 (1.9)	13 (1.5)
**C (Cardiovascular system)**	**2 (3.8)**	**76 (8.6)**
C01 (Cardiac therapy)	1 (1.9)	6 (0.7)
C02 (Antihypertensives)	-	10 (1.1)
C07 (Beta blocking agents)	-	2 (0.2)
C08 (Calcium channel blockers)	-	4 (0.5)
C09 (Agents acting on the renin-angiotensin system)	-	14 (1.6)
C10 (Lipid modifying agents)	1 (1.9)	40 (4.5)
**D (Dermatologicals)**	**-**	**13 (1.5)**
D01 (Antifungals for dermatological use)	-	7 (0.8)
D05 (Antipsoriatics)	-	2 (0.2)
D10 (Anti-acne preparations)	-	3 (0.3)
D11 (Other dermatological preparations)	-	1 (0.1)
**G (Genito urinary system and sex hormones)**	**1 (1.9)**	**36 (4.1)**
G01 (Gynecological antiinfectives and antiseptics)	-	1 (0.1)
G03 (Sex hormones and modulators of the genital system)	1 (1.9)	33(3.8)
G04 (Urologicals)	-	2 (0.2)
**H (Systemic hormonal preparations)***	**1 (1.9)**	**21 (2.4)**
H02 (Corticosteroids for systemic use)	-	5 (0.6)
H03 (Thyroid therapy)	1 (1.9)	16 (1.8)
**J (Anti-infectives for systemic use)**	**21 (40)**	**302 (34)**
J01 (Antibacterials for systemic use)	13 (25)	233 (26)
J02 (Antimycotics for systemic use)	-	6 (0.7)
J04 (Antimycobacterials)	7 (13)	60 (6.8)
J05 (Antivirals for systemic use)	1 (1.9)	3 (0.3)
**L (Antineoplastic and immunomodulating agents)**	**1 (1.9)**	**87 (9.9)**
L01 (Antineoplastic agents)	1 (1.9)	38 (4.3)
L02 (Endocrine therapy)	-	19 (2.2)
L03 (Immunostimulants)	-	4 (0.5)
L04 (Immunosuppressants)	-	26 (3.0)
**M (Musculo-skeletal system)**	**6 (11)**	**113 (13)**
M01 (Antiinflammatory and antirheumatic products)	2 (3.8)	100 (11)
M02 (Topical products for joint and muscular pain)	-	4 (0.5)
M03 (Muscle relaxants)	-	7 (0.8)
M04 (Antigout preparations)	4 (7.6)	2 (0.2)
**N (Nervous system)**	**17 (32)**	**76 (8.6)**
N02 (Analgesics)	-	3 (0.3)
N03 (Antiepileptics)	15 (28)	29 (3.3)
N05 (Psycholeptics)	2 (3.8)	14 (1.6)
N06 (Psychoanaleptics)	-	23 (2.6)
N07 (Other nervous system drugs)	-	7 (0.8)

**P (Antiparasitic products, insecticides, and repellents)**	**-**	**10 (1.1)**
P01 (Antiprotozoals)	-	1 (0.1)
P02 (Anthelmintics)	-	9 (1.0)
**R (Respiratory system)**	**-**	**6 (0.7)**
R02 (Throat preparations)	-	1 (0.1)
R03 (Drugs for obstructive airway diseases)	-	3 (0.3)
R06 (Antihistamines for systemic use)	-	2 (0.2)
**S (Sensory organs)**	-	**3 (0.3)**
S01 (Ophthalmologicals)	**-**	3 (0.3)
**Herbal and dietary supplements**	**-**	**53 (6.0)**
**Anabolic and androgenic steroids**	**-**	**36 (4.1)**

ATC: Anatomic Therapeutic Classification; DILI: drug-induced liver injury; DRESS: Drug Reaction with Eosinophilia and Systemic Symptoms.

* Excluding sex hormones and insulins.
